# Antiparasitic Activity of *Hedera helix* Extract-Loaded Chitosan Nanoparticles in Experimentally Induced Giardiasis

**DOI:** 10.3390/vetsci13020207

**Published:** 2026-02-22

**Authors:** Hany M. El-Wahsh, Faten Abdullah Al Braikan, Doaa Naguib, Suzan Awad Abdel Ghany Morsy, Alshaymaa M. Abdelmenem, Shaimaa G. Ibrahim, Hebatallah Husseini Atteia, Hend Mohamed Hussein, Mohammad Mousa Alshumrani, Ashraf Fawzy Mosa Ahmed, Mariham George Loqa, Ahlam F. Moharam

**Affiliations:** 1 Marine Biology Department, Faculty of Marine Sciences, King Abdulaziz University, Jeddah 21589, Saudi Arabia; hmelwahsh@kau.edu.sa; 2 Department of Clinical Microbiology and Immunology, Faculty of Medicine, King Abdulaziz University, Jeddah 21589, Saudi Arabia; falbraikan@kau.edu.sa; 3 Department of Hygiene and Zoonoses, Faculty of Veterinary Medicine, Mansoura University, Mansoura 35516, Egypt; 4 Department of Clinical Pharmacology, Faculty of Medicine, Alexandria University, Dr. Fahmi Abdelmeguid St., Mowassah Campus, Alexandria 21561, Egypt; susan.ulghani@alexmed.edu.eg; 5 MBBS Program, Pathological Sciences Department, Fakeeh College for Medical Sciences, Jeddah 21461, Saudi Arabia; 6 Department of Histology and Cell Biology, Faculty of Medicine, Fayoum University, Fayoum 63511, Egypt; sma25@fayoum.edu.eg (A.M.A.);; 7 Department of Pharmacology and Toxicology, Faculty of Pharmacy, October 6 University, Giza 12566, Egypt; drshaimaa.gomaa@yahoo.com; 8 Department of Pharmaceutical Chemistry, Faculty of Pharmacy, University of Tabuk, Tabuk 71491, Saudi Arabia; hatteia@ut.edu.sa; 9 Department of Biochemistry, Faculty of Pharmacy, Zagazig University, Zagazig 44519, Egypt; 10 Department of Parasitology, Faculty of Medicine, Ain Shams University, Cairo 11566, Egypt; d.hend_m@yahoo.com; 11 Al Rayan National College of Medicine, Al-Madinah 41411, Saudi Arabia; 12 Department of Psychology, Taif University, Taif 21944, Saudi Arabia; shumrani@tu.edu.sa; 13 Molecular and Applied Parasitology, Medical Research Institute, Alexandria University, Alexandria 21561, Egypt; ashraf_fawzy95@yahoo.com; 14 Department of Parasitology, Faculty of Medicine, Benha University, Benha 13518, Egypt; ahlam.moharam@fmed.bu.edu.eg

**Keywords:** giardiasis, ivy, *Hedera helix* leaf extract (HLE), chitosan, nanoparticles, metronidazole, zoonoses

## Abstract

Giardiasis, caused by *Giardia duodenalis*, is a common gastrointestinal infection. This study aimed to evaluate the effects of *Hedera helix* leaf extract (HLE) and HLE-loaded chitosan nanoparticles (HLE-CsNPs) against *Giardia duodenalis* isolates from individuals with gastrointestinal issues, using an experimental rat model. Stool samples from 147 participants were analyzed for *Giardia duodenalis*, with positive samples further characterized by nested PCR-RFLP, revealing a 4.8% prevalence, all belonging to assemblage B. Ten groups of male albino rats were used to evaluate the antigiardial activity of various treatments. This included five non-infected groups [one untreated and four treated with HLE, HLE-CsNPs, CsNPs, and metronidazole (MTZ)] and five infected groups [one untreated and four similarly treated]. Treatment efficacy was assessed using parasite counts, intestinal histopathology, CD117 immunohistochemistry, and markers of liver and kidney function. HLE-CsNPs markedly reduced *Giardia* cysts by 88.8%, approaching the 99.2% reduction achieved by MTZ, while also improving intestinal architecture and reducing inflammation. Importantly, HLE and HLE-CsNPs provided superior protection for the liver and kidneys compared to MTZ. In conclusion, HLE-CsNPs exhibited significant antigiardial activity and organ protection in rats, suggesting a potential alternative treatment for *Giardia duodenalis* isolated from individuals with gastrointestinal issues.

## Simple Summary

Giardiasis is a reemerging protozoal zoonotic disease that affects both humans and animals and continues to be a public health concern worldwide. Treatment typically relies on metronidazole; however, this drug can cause side effects and treatment failures in some cases. In this study, we tested ivy (*Hedera helix*) leaf extract and *Hedera helix* extract loaded into chitosan nanoparticles as alternative treatments for *Giardia* spp. Both treatments significantly reduced the number of parasites and improved the structure of intestinal tissues. Additionally, they decreased inflammatory cell activity, as indicated by reduced expression of CD117. Importantly, unlike metronidazole, both *Hedera helix* extract and *Hedera helix* extract-loaded chitosan nanoparticles offered protection to the liver and kidneys from damage. Our findings suggest that *Hedera helix* leaf extract, particularly in nanoparticle form, is a potential alternative therapy for giardiasis.

## 1. Introduction

Giardiasis is an infection caused by the protozoan *Giardia duodenalis*, also known as *Giardia intestinalis* or *Giardia lamblia*. This zoonotic, flagellated parasite infects the digestive tracts of humans and animals, making it one of the most common parasites in mammals [1,2]. Infection can occur through direct contact with infected individuals or animals, or by consuming contaminated food or water that contains environmentally resistant cysts [3,4]. Giardiasis is a reemerging zoonotic disease that poses a significant public health threat globally [5]. It leads to around 280 million cases of diarrhea annually, imposing a major health and economic burden [6,7]. In addition to common symptoms such as diarrhea, flatulence, abdominal pain, fever, and bloating, giardiasis may also cause weight loss and failure to thrive [8]. 

*G. duodenalis* includes at least eight genetic subgroups (assemblages A–H), each with specific host preferences. Zoonotic assemblages A and B are found in humans and various mammals, while assemblages C and D are present in canids, E in hoofed animals, F in cats, G in rodents (particularly rats), and H in pinnipeds [1]. Research shows that *G. duodenalis* can be transmitted by rodents [9]. Species like *G. muris*, *G. microti*, and *G. duodenalis* (assemblages G and B) infect these animals [10]. Experimental infections in mice and gerbils with zoonotic assemblages are possible [11], and there are rare cases of such infections in the wild [12]. Recent diagnostic methods include C-kit (CD117) immunostaining, which effectively identifies *G. duodenalis* in duodenal biopsies by highlighting its paired nuclei, aiding in diagnosis [13].

As there is currently no safe and effective vaccine for giardiasis, management strategies primarily focus on pharmacological treatments [14]. The standard therapy for giardiasis is metronidazole (MTZ), tinidazole, furazolidone, and albendazole [15], which carry a high risk of numerous side effects like headache, vertigo, nausea, and a metallic taste, in addition to the severe neurological complications [16,17], and the risk of mutagenesis and carcinogenesis recognized in animal models with exposure to MTZ for long durations or with high doses [18,19,20]. There is increasing evidence of treatment failures and emerging drug resistance. Studies indicate that treatment failures linked to clinical resistance can be as high as 70% of cases [21,22].

As a result, recent research has shifted towards exploring herbal medicine and plant-derived products, which may offer safer substitutes for the treatment of various diseases compared to synthetic drugs [23]. One commonly used plant in traditional medicine is ivy leaves, known as “English Ivy” or “*Hedera helix*.” This plant grows throughout Europe and is frequently utilized for treating respiratory disorders [24,25,26]. Research has proven that it can be used as an expectorant [27], bronchodilator [28], antioxidant [29], and an anti-inflammatory agent [30]. Moreover, recent studies suggested its potential antimicrobial action [31,32]. However, one significant challenge to the use of Ivy leaves is their poor oral bioavailability, which ranges from approximately 0.118% to 0.250% [33]. To address this issue, researchers are increasingly exploring nanotechnology as a method to enhance the efficacy of drugs by improving their bioavailability [34].

Nanoparticles (NPs) are increasingly utilized in both medicine and industry, as they enhance drug delivery and minimize side effects [35,36]. They improve bioavailability, solubility, and toxicity profiles, making them essential in pharmaceuticals [37]. Chitosan nanoparticles, in particular, are gaining attention for their role in immobilization and controlled release, which are crucial for targeted drug delivery in new projects [38]. Studies have demonstrated the effectiveness of chitosan nanoparticles in steroid and enzymatic immobilization, as well as in delivering siRNA [39]. As a result, chitosan nanoparticles are proposed as optimal carriers for antibacterial [40], antifungal [41], and anti-parasitic [42] treatments. They have shown remarkable inhibitory effects against parasitic infections by inhibiting and lysing cell walls, suppressing protein synthesis, altering cell membranes, inhibiting nucleic acid synthesis, and exhibiting antimetabolite activity [43]. Therefore, in this study, we aimed to evaluate the efficacy of *Hedera helix* leaf extract-loaded chitosan nanoparticles (HLE-CsNPs) against *Giardia duodenalis* isolates obtained from individuals with gastrointestinal complaints, using an experimental rat model.

## 2. Materials and Methods

### 2.1. Ethical Consideration

This study was conducted at the Medical Research Institute of Alexandria University in Egypt, with approval from the Ethics Committee of the Faculty of Pharmacy at October 6 University in Cairo, Egypt (Approval Number: PRE-Ph-2312004). All methodologies were transparently reported in line with ARRIVE guidelines, upholding high ethical research standards. The survey followed International Ethical Guidelines for Research Involving Human Subjects, with informed consent obtained from parents or legal guardians of participating children. The use of human-derived isolates was justified by their relevance for evaluating cross-host infectivity and therapeutic efficacy under controlled conditions.

### 2.2. Detection of Giardia duodenalis

#### 2.2.1. Microscopic Examination of Stool Samples

*G. duodenalis* was isolated from fresh stool samples collected from 147 individuals attending the outpatient clinic of the parasitology department, Medical Research Institute of Alexandria University. The participants included 105 non-diarrheic patients and 42 diarrheic ones. The cohort comprised 78 females and 69 males, with ages ranging from 10 to 60 years. No a priori sample size calculation was conducted, as this epidemiological survey was exploratory and depended on the availability of clinical samples during the study period. All samples were processed and examined microscopically. Wet mounts were prepared from freshly passed stool using direct saline (NaCl 0.9%) and Lugol’s iodine. For the saline wet mount, a small amount of stool was emulsified in a drop of saline on a clean glass slide and then covered with a coverslip. In the iodine mount, Lugol’s iodine solution was mixed with an equal volume of stool. Each slide was systematically inspected under low (10×) and high (40×) magnification, and notes were taken regarding the presence and appearance of *Giardia* cysts and trophozoites. Accurate parasite stage detection and differentiation were made possible by this dual staining method, which reduced false negatives [44,45].

#### 2.2.2. DNA Extraction

The positive microscopic samples containing purified *G. duodenalis* were washed with PBS for 5 min, followed by centrifugation at 500× *g* for 5 min. After discarding the supernatant, the resulting pellets underwent a thermal shock to facilitate genomic DNA extraction using the QIAamp^®^ DNA Stool Mini Kit (Qiagen, Hilden, Germany), in accordance with the manufacturer’s guidelines. The success of the extraction was confirmed through gel electrophoresis, and the purity of the DNA was assessed using a Nanodrop spectrophotometer. The extracted DNA was either used immediately or stored at −20 °C for future use.

#### 2.2.3. PCR Amplification and RFLP Assay

Isolated DNA was amplified using nested PCR with Thermo Scientific DreamTaq™ Green PCR Master Mix 2× (Thermo Fisher Scientific, K1081, Loughborough, UK), employing a set of previously published primer pairs targeting the β-giardin gene, synthesized by Invitrogen, Thermo Fisher Scientific, Frederick, MD, USA (Table S1). Amplification was carried out using the Taq DNA polymerase included in the master mix under control conditions. The PCR products were separated by electrophoresis on a 1.5% agarose gel (Sigma-Aldrich, St. Louis, MO, USA) and visualized using ethidium bromide staining (Sigma-Aldrich, St. Louis, MO, USA) under UV illumination to identify positive samples. A *Giardia duodenalis*-positive sample was genetically characterized using nested-PCR-RFLP. The nested-PCR amplification products were then digested with the Hae-III endonuclease enzyme from New England Biolabs Inc. (Ipswich, MA, USA), following the manufacturer’s guidelines. The resulting restriction fragments were examined further. A second round of agarose gel electrophoresis (1.5%) was applied to separate the restriction fragments into representative bands of *Giardia duodenalis* subtypes. These fragments were directly stained with ethidium bromide and visualized under UV transillumination to define *Giardia duodenalis* assemblages.

### 2.3. Animals

This study involved 120 male albino rats, each weighing approximately 200 ± 10 g. Initially, the rats were examined daily to ensure that they were free of parasitic infections. Throughout the experiment, the rats were provided with a standard diet and had unrestricted access to water. They were housed six per cage in the animal care facilities at the Medical Research Institute, Alexandria University [46].

### 2.4. Drugs and Chemicals

#### 2.4.1. Metronidazole (MTZ)

Metronidazole (Flagyl; suspension of 125 mg/5 mL, SANOFI. Co., Cairo, Egypt) was given orally at a dose of 7 mg/rat/day for 7 consecutive days [47].

#### 2.4.2. *Hedera helix* Leaf Extract (HLE)

We purchased fresh and healthy *Hedera helix* leaves from E.G.P.I Co., Giza, Egypt. The leaves are carefully collected and thoroughly washed first with tap water, then with distilled water, to remove any debris and contaminants. After washing, the leaves are shade-dried at room temperature (25–30 °C) for 7–10 days until they reach a constant weight. Once dried, the leaves are ground into a fine powder using a laboratory grinder and stored in airtight containers at 4 °C until extraction. Fifty to one hundred grams of leaf powder are mixed with distilled water at a ratio of 1:10 (*w*/*v*). This mixture is then heated to a temperature of 60–80 °C for 30 to 60 min while being continuously stirred. After cooling, the extract is filtered through Whatman No. 1 filter paper. The resulting filtrate can be concentrated using a rotary evaporator or freeze-dried to obtain a dry extract, which is then stored at 4 °C [23].

#### 2.4.3. Chitosan

Chitosan with a deacetylation grade of 93% and other chemicals were purchased from Sigma Aldrich Chemical Co. (St. Louis, MO, USA).

### 2.5. Nanoparticle Preparation and Characterization

#### 2.5.1. Preparation of CsNPs

The ionotropic gelation method, a widely used technique that employs an ionic cross-linker to enhance the formation of nanoparticles, was utilized to synthesize chitosan nanoparticles (CsNPs). The ionic cross-linker used in this process was tripolyphosphate (TPP). To create a coacervate complex, two aqueous solutions were mixed: one containing the polyanion TPP and the other containing the chitosan polymer. The chitosan was dissolved in a 1% (*v*/*v*) acetic acid solution while continuously stirred by a magnetic stirrer at room temperature to facilitate the formation of the nanoparticles. Next, the TPP solution was added dropwise while maintaining constant stirring, resulting in the spontaneous formation of nanoparticles. The mixture developed a milky appearance, indicating nanoparticle development. After several hours of stirring to stabilize the nanoparticles, they were recovered through ultracentrifugation for 30 min at 10,000 rpm. The precipitated nanoparticles were then washed with distilled water and freeze-dried for later use. To ensure stability, the dried CsNPs were stored at 4 °C [48].

#### 2.5.2. Preparation of *Hedera helix* Extract-Loaded Chitosan Nanoparticles (HLE-CsNPs)

An aqueous extract of *Hedera helix* was prepared at a concentration of 1 mg/mL to load the leaf extract onto chitosan nanoparticles (CsNPs). To facilitate adsorption and encapsulation, this extract was combined with chitosan nanoparticles at a concentration of 20 mg/mL and stirred continuously for a full day. Afterward, the mixture was centrifuged at 10,000 rpm to separate the extract-containing nanoparticles. The nanoparticles were rinsed with distilled water to remove any remaining extract and then centrifuged again. The resulting *Hedera helix*-loaded chitosan nanoparticles (HLE-CsNPs) were dried and stored for later analysis and use [49].

#### 2.5.3. Nanoparticle Characterization

The physicochemical properties of CsNPs and HLE-CsNPs were characterized through the measurement of zeta potential and particle size. Additionally, morphological analysis of the shape and surface structure of the nanoparticles was conducted using transmission electron microscopy (TEM). To confirm the successful encapsulation of HLE-CsNPs, Fourier Transform Infrared Spectroscopy (FTIR) was performed to detect the relevant functional groups [50].

### 2.6. G. duodenalis Cyst Inoculum Preparation

*Giardia duodenalis* cysts used in this study were isolated from stool samples of human patients with giardiasis and genetically characterized before experimentation. The purified cysts were prepared into an inoculum for orally infecting rats, ensuring consistent infectivity. To concentrate the cysts, samples were centrifuged three times at 2000 rpm for five minutes, with saline washes in between. After the final wash, regular saline (NaCl 0.9%) was added to the sediment. The concentration of *G. duodenalis* cysts in 1 mL of the sediment was measured using a hemocytometer, and the counts were averaged from three measurements to achieve a targeted concentration of 10^4^ cysts/mL. Rats in the non-infected groups were administered 1 mL of regular saline (NaCl 0.9%), while those in the infected groups received 1 mL of the suspension containing 10^4^ cysts orally using gastric gavage [47]. Starting on the third day post-infection (P.I.), each rat underwent three separate stool examinations to confirm the infection, using both Lugol’s iodine-stained and unstained smears [44]. The treatments began on the eighth day after the infection. Before treatment, fecal cyst counts were comparable across all infected groups before treatment, establishing a uniform infection for assessing treatment efficacy. The infected non-treated group’s cyst output served as the baseline for calculating the percentage reduction in the treated groups.

### 2.7. Experimental Design

A total of 120 male albino rats were utilized, divided into the following groups (12 rats per group, with two replicates for each):

Group I: Non-infected non-treated.

Group II: Infected non-treated

Group III (Infected + MTZ-treated): The infected group was treated with metronidazole orally via gastric gavage at a dose of 7 mg/rat/day for 7 consecutive days [46].

Group IV (Infected + HLE-treated): The infected group was treated with aqueous extract of *Hedera helix* orally via gastric gavage at a dose of 200 mg/kg for 7 consecutive days [51].

Group V (Infected + HLE-CsNPs-treated): The infected group was treated with *Hedera helix* aqueous extract-loaded CsNPs orally via gastric gavage at a dose of 200 mg/kg for 7 consecutive days.

Group VI (infected + CsNPs treated): The infected group was treated with chitosan nanoparticles suspension orally in a dose of (50 μg/day) via gastric gavage for 7 consecutive days [47].

Group VII (Non-infected + HLE-treated): The non-infected group was treated with aqueous extract of *Hedera helix* orally via gastric gavage at a dose of 200 mg/kg for 7 consecutive days.

Group VIII (Non-infected + HLE-CsNPs-treated): The non-infected group was treated with *Hedera helix* aqueous extract-loaded CsNPs orally via gastric gavage at a dose of 200 mg/kg for 7 consecutive days.

Group IX (Non-infected + CsNPs treated): The non-infected group was treated with chitosan nanoparticles suspension orally in a dose of (50 μg/day) via gastric gavage for 7 consecutive days.

Group X (Non-infected + MTZ-treated): The non-infected group was treated with metronidazole orally via gastric gavage at a dose of 7 mg/rat/day for 7 consecutive days.

The treatment lasted for seven days. Fecal samples for the final cyst count were collected on the eighth day after the treatment began. On the ninth day post-treatment, all rats were humanely euthanized with isoflurane anesthesia. Blood and small intestine samples were collected for analysis, with blood drawn from the abdominal artery in plain tubes, allowed to clot at room temperature, and centrifuged to obtain serum for the assessment of hepatic and renal function parameters.

### 2.8. G. duodenalis Cyst Counting in the Infected Groups

After the treatment period ended, three stool samples were collected from each rat in sterile tubes. The samples were combined, and one gram of this mixture was emulsified in saline (NaCl 0.9%) and centrifuged at 2000 rpm for two minutes. The sediment was then examined for parasites, and the supernatant was discarded. A hemocytometer was used to analyze ten high-power fields (40×) for cyst counts, and the average number of cysts per field was calculated [46].

### 2.9. Biochemical Analysis

Serum samples were analyzed for serum glutamic oxaloacetic transaminase (SGOT) and serum glutamic pyruvic transaminase (SGPT) to assess liver function, using kits from Biosystems Co. (Barcelona, Spain). Additionally, creatinine and urea levels were measured to evaluate kidney function, following the modified methods of Jaffé and Berthelot, respectively [52,53]. The specific values measured included SGPT (Spectrum Co., Stamford, CT, USA EDA Reg.: R02LIVD23V1), SGOT (Spectrum Co., EDA Reg.: R02LIVD23V1), Creatinine (Vitro Scientific Co., Cairo, Egypt, REF: 11102, LOT: 111069), and Urea (Biomed Diagnostic Co., HongKong, China LOT: 18522).

### 2.10. Histopathological Analysis

After the completion of treatment, the small intestine from each sacrificed rat was dissected and preserved in 10% neutral buffered formalin (NBF) for a full day. Following the methods outlined by Bancroft and Layton [54]. The samples were dehydrated using a graded ethanol series (ranging from 70% to 100%), cleared in xylene, and then embedded in metal molds filled with paraffin. Serial sections of 5 µm thickness were obtained using microtomy. These sections were mounted on glass slides and stained directly with hematoxylin and eosin to observe intestinal histopathological changes. Additionally, a Periodic Acid-Schiff (PAS) reaction was performed to evaluate goblet cells and the intestinal brush border, as positive reactions are indicative of the presence of carbohydrates, particularly glycogen, as well as monosaccharides, polysaccharides, glycoproteins, and mucoproteins.

### 2.11. Immunohistochemical Staining Using CD117/c-Kit Antibody

The paraffin-fixed intestinal sections underwent immunohistochemistry (IHC) using the peroxidase-labeled streptavidin-biotin method. A rabbit polyclonal antibody (ABclonal Inc., Woburn, MA, USA, Catalog No.: A0357) was employed to identify CD117 protein in the intestinal tissues, which helped demonstrate the presence of *Giardia* trophozoites and intraepithelial lymphocytes. Peroxidase blocking solutions were applied to suppress endogenous tissue peroxidase activity. For antigen retrieval, citrate buffer solutions were utilized, and the microwave was set to 90 °C for nine minutes. After incubating the diluted primary antibodies at a 1:200 ratio, the tissues were left at room temperature overnight. The addition of diaminobenzidine (DAB) facilitated coloration and visualization. Mayer’s hematoxylin served as a counterstain for the nuclei, providing good contrast. The kidney was used as the positive control, as recommended by the manufacturer. The CD117 reaction was cytoplasmic, and a negative control was performed by omitting the primary antibody to evaluate any nonspecific binding of the secondary antibody [13].

### 2.12. Quantitative Morphometric Analysis

A light microscope (Olympus BX43F, Tokyo, Japan) was used for the initial visualization of images. The mean thickness of the muscularis externa (ME) in H&E-stained sections was assessed at a magnification of ×400. Additionally, the mean area percentage of PAS reaction in PAS-stained sections was evaluated at ×200, and the mean area percentage of CD117 in immunohistochemical sections was analyzed at ×400. These histological parameters were quantitatively assessed using the “Toup View” image analyzer computer system (China) at the Histology Department, Faculty of Medicine, Fayoum University. Measurements were performed by analyzing ten randomly selected non-overlapping fields per slide. A representative slide was evaluated for each animal, resulting in a total of ten fields per animal. The fields were systematically chosen from the same region to ensure consistency while avoiding overlaps and artifacts.

### 2.13. Statistical Analysis

The data were analyzed using GraphPad Prism software version 10 and SPSS software package version 22. To assess the efficacy of the treatments, we compared the five infected groups (infected untreated, infected treated with HLE, infected treated with HLE-CsNPs, infected treated with CsNPs, and infected treated with MTZ). A one-way ANOVA was used for the initial comparison, followed by pairwise comparisons with the post hoc Tukey test. To determine whether the continuous data followed a normal distribution, the Shapiro–Wilk test was utilized. The quantitative data are presented as mean ± standard deviation. A *p*-value of less than 0.05 was considered statistically significant.

## 3. Results

### 3.1. Prevalence of Giardia duodenalis Infections and the Assemblages Identified from Stool Samples of Surveyed Individuals (n = 147)

*Giardia duodenalis* was detected in 7 out of 147 stool samples (4.8%) through microscopy, and all positive samples were confirmed as assemblage B using nested PCR-RFLP (Figure S1). The prevalence of the infection was not statistically significant (*p* = 0.84) across different age groups: 5% in individuals aged 10–30 years and 4.5% in those aged 31–60 years. Additionally, there was no significant difference in infection rates between males (4.3%) and females (5.1%), as well as among individuals with diarrhea (4.8%) and those without (4.8%) (*p* = 0.72 and *p* = 0.89, respectively) (Table S2).

### 3.2. Nanoparticle Physicochemical Characterization

#### 3.2.1. Zeta Potential

**Figure 1:**
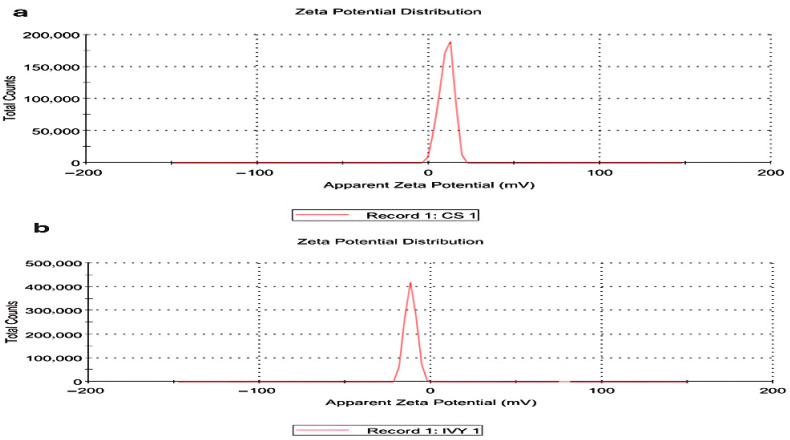
Zeta potential distribution. (**a**) Zeta potential distribution of CsNPs. (**b**) Zeta potential distribution of HLE-CsNPs. The CsNPs exhibit a peak just after zero, while the HLE-CsNPs display a peak just before zero, indicating differences in surface charge and stability between the two formulations. CsNPs: chitosan nanoparticles, HLE-CsNPs: *Hedera helix* extract-loaded chitosan nanoparticles.

The zeta potential measurements of the nanoparticles indicated that the CsNPs and HLE-CsNPs have different charge distributions. The CsNPs displayed a peak just after zero, reaching a maximum count of approximately 20,000. In contrast, the HLE-CsNPs showed a peak just before zero, with a higher intensity exceeding 40,000. The peak just after zero suggests that the CsNPs possess a positive zeta potential. Conversely, the HLE-CsNPs display a peak just before zero, indicating a shift toward a zeta potential that is more neutral or slightly negative (Figure 1).

#### 3.2.2. Transmission Electron Microscopy (TEM) Analysis

**Figure 2:**
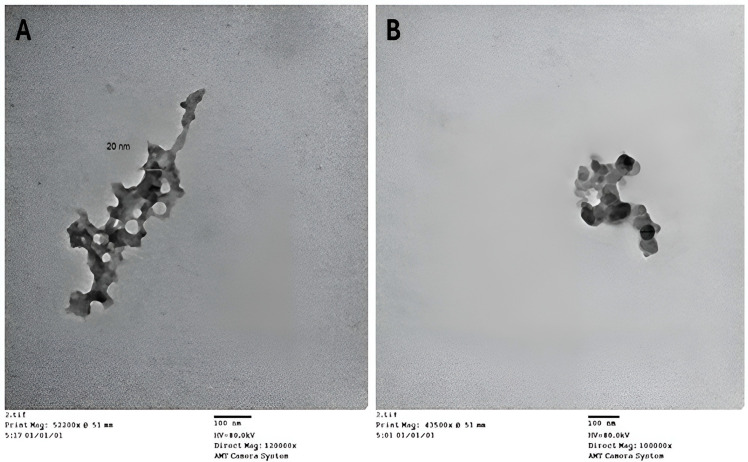
TEM images. (**A**) TEM image of CsNPs. (**B**) TEM image of HLE-CsNPs. The images were captured using an AMT Camera System at magnifications of 120,000× and 100,000×, respectively. The scale bar represents 100 nm. TEM: transmission electron microscopy, CsNPs: chitosan nanoparticles, HLE-CsNPs: *Hedra helix* leaf extract loaded onto chitosan nanoparticles.

TEM was utilized to measure the size distribution and examine the morphology of the synthesized chitosan nanoparticles (CsNPs) and HLE-loaded CsNPs (HLE-CsNPs). The images indicate that the nanoparticles exhibit a spherical shape and a uniform size distribution. In the case of CsNPs (Figure 2A), the particles appear to be evenly distributed and possess a smooth surface. Some aggregation was noted, which may be attributed to the drying procedure used to prepare the sample. The average particle size was found to be between 20 and 50 nm, aligning with previous studies that reported chitosan-based nanoparticles produced by ionic gelation to have sizes ranging from 10 to 80 nm [48]. HLE-CsNPs exhibited a greater tendency to aggregate (Figure 2B), while the nanoparticles remained within the nanoscale range of 30–50 nm. This indicates that the plant extract was successfully loaded without significantly altering its shape (Figure 2).

#### 3.2.3. Fourier Transform Infrared (FTIR) Analysis

**Figure 3:**
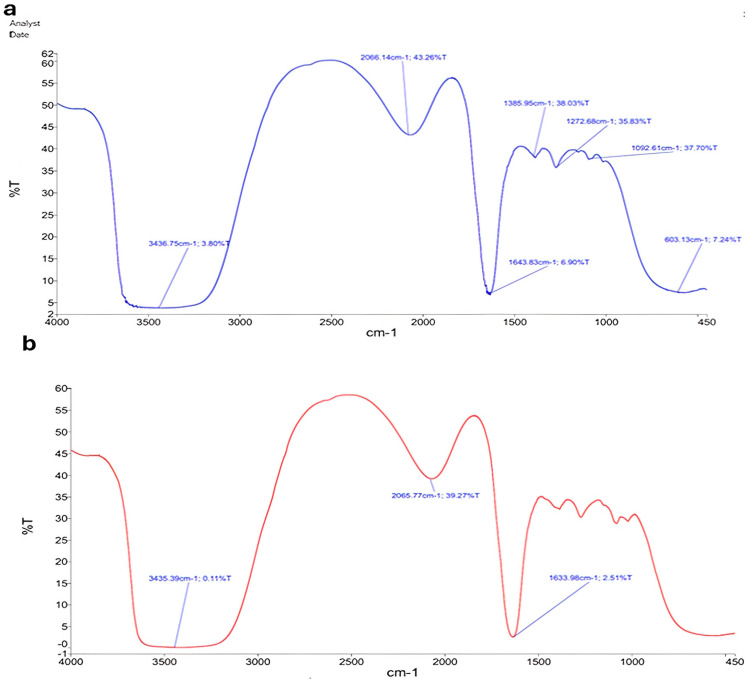
FTIR spectrum. (**a**) FTIR spectrum of the CsNPs shows characteristic peaks at 3435.75 cm^−1^, 2066.14 m^−1^, 1358.95 cm^−1^, 1272.68 cm^−1^, 1092.61 cm^−1^, and 603.13 cm^−1^. (**b**) FTIR spectrum of the HLE-CsNPs shows characteristic peaks at 3435.39 cm^−1^, 2065.77 cm^−1^, and 1633.98 cm^−1^. FTIR: Fourier transform infrared, CsNPs: Chitosan nanoparticles, HLE-CsNPs: *Hedra helix* Extract Chitosan nanoparticles.

The FTIR spectra of CsNPs showed distinctive peaks at 3435.75 cm^−1^, 2066.14 cm^−1^, 1358.95 cm^−1^, 1272.68 cm^−1^, 1092.61 cm^−1^, and 603.13 cm^−1^ (Figure 3a). The broad absorption band at 3435.75 cm^−1^ indicates the presence of hydroxyl groups in chitosan, as it corresponds to O-H stretching vibrations. At 2066.14 cm^−1^, the peak denotes C≡C or C≡N stretching. Peaks at 1358.95 cm^−1^ and 1272.68 cm^−1^ represent C-H bending and C-N stretching, respectively, while peak 1092.61 cm^−1^ is linked to C-O stretching. The band at 603.13 cm^−1^ represents out-of-plane bending vibrations. Characteristic peaks were observed at 3435.39 cm^−1^, 2065.77 cm^−1^, and 1633.98 cm^−1^ for HLE-CsNPs (Figure 3b). The FTIR spectrum of CsNPs showed characteristic peaks corresponding to hydroxyl, amine, and polysaccharide functional groups. In HLE-CsNPs, a similar pattern was observed with slight shifts and the appearance of a new band at 1633.98 cm^−1^. This band, attributed to carbonyl or amide groups, indicates interactions between chitosan and HLE constituents (e.g., polyphenols and flavonoids). These spectral changes confirm that HLE bioactive compounds were successfully incorporated into the chitosan nanoparticles, likely through hydrogen bonding, electrostatic interactions, or covalent linkages. This highlights the effective loading of the extract and potential structural modification of the nanoparticles. The presence of this peak suggests that HLE bioactive compounds have interacted with chitosan, likely through hydrogen bonding, electrostatic interactions, or possible Schiff base (imine) formation between chitosan’s amine groups and carbonyl-containing components in HLE. This confirms the successful loading of HLE into the CsNPs and suggests potential structural modifications in the nanoparticle formulation.

### 3.3. Effect of Treatment on G. duodenalis Cyst Count per Gram of Feces and the Percentage Reduction in Infection

The assessment of the number of *Giardia duodenalis* cysts per gram of feces showed a statistically significant (*p* < 0.001) reduction in all treated infected groups compared to the untreated infected group. The greatest improvement was observed in the infected + MTZ-treated group, followed by the infected + HLE-CsNPs-treated and the infected + HLE-treated groups (Table 1). While there was a variation in the reduction rates among the different groups treated with HLE, HLE-CsNPs, and CsNPs, these differences were not statistically significant (*p* > 0.05). The results indicate that treatment with HLE-CsNPs or HLE alone achieved a high reduction rate of 88.8% and 78.7%, respectively, compared to MTZ, which achieved a 99.17% reduction (Table 1).

### 3.4. Biochemical Assessment

#### 3.4.1. Liver Function Tests

The serum enzymatic activities of SGPT and SGOT showed a significant increase in the infected + MTZ-treated and non-infected + MTZ-treated groups compared to the non-infected non-treated group (*p* = 0.003 and *p* = 0.005, respectively). This indicates that MTZ treatment resulted in substantial liver stress or damage. In contrast, administering HLE-CsNPs to the infected group for 7 days resulted in SGPT and SGOT levels similar to those in the non-infected non-treated group (Table 2).

#### 3.4.2. Renal Function Tests

The results showed a highly significant increase in creatinine levels in the infected + MTZ-treated group compared to the non-infected non-treated group (*p* = 0.003). This indicates that MTZ caused renal impairment. Similarly, the infected + HLE-treated group exhibited a significant increase in creatinine levels (*p* = 0.004), suggesting a mild effect on renal function. However, no significant difference in serum creatinine levels was observed in the infected + HLE-CsNPs-treated group compared with the non-infected non-treated group (*p* = 0.72) or the infected non-treated group (*p* = 0.26) (Table 2). A significant increase in serum urea levels was observed in both the infected + MTZ-treated group and the non-infected + MTZ-treated group compared to the non-infected non-treated group (*p* = 0.001 and *p* = 0.002, respectively). In contrast, the infected + HLE-CsNPs-treated group exhibited a significant decrease in serum urea levels (*p* = 0.001) (Table 2).

### 3.5. Histopathological Results

#### 3.5.1. Histological Examination of H&E-Stained Sections from the Small Intestine

**Figure 4:**
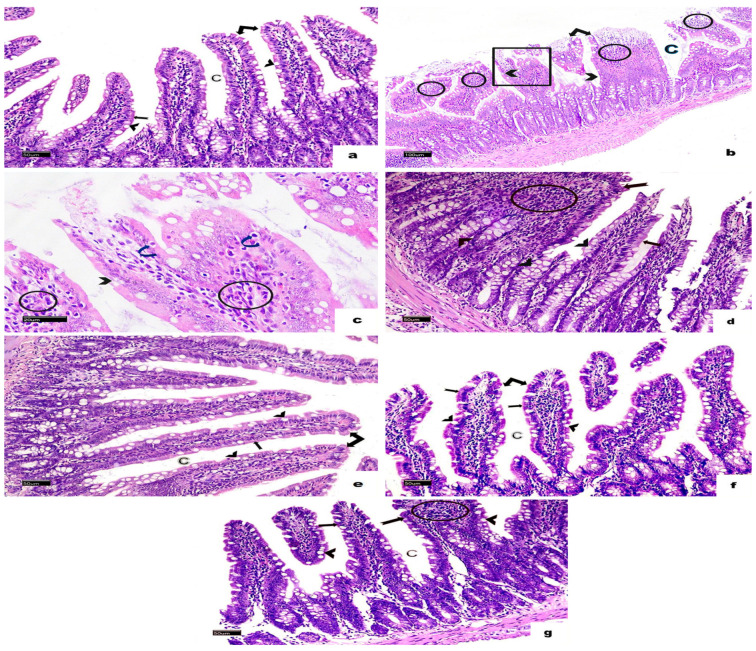
Light photomicrographs of a section in the small intestine of rats stained with H&E in all experimental groups. (**a**) The non-infected, non-treated group shows normal villi (double arrow) and crypt pattern (C). The villus appears with a brush border (bifid arrow), and many goblet cells (arrowheads). (**b**) The infected non-treated group shows disturbed crypts (C), and some villi appeared short, fused, and flattened (square), while others were ulcerated (double arrow). Marked mononuclear cellular infiltration (circles) and many disturbed goblet cells (arrowheads) are also noticed. (**c**) A higher magnification of the same photomicrograph shows small dark nuclei in the cells lining the villi (curved arrows), disturbed goblet cells (arrowhead), and mononuclear cellular infiltration (circles). (**d**) Most villi in the sections of the infected + MTZ-treated group restore their normal structure (arrow), a few of them appear broad (bifid arrow) with mononuclear inflammatory cell infiltration (circle), and many goblet cells (arrowheads). (**e**) The infected + HLE-treated group and (**f**) The infected + HLE-CsNPs-treated group show normal villi (double arrow) with a brush border (bifid arrows) and crypts (C) with many goblet cells (arrowheads). (**g**) The infected + CsNPs-treated group most villi appeared normal (arrow), with mononuclear cellular infiltration (circle) in another broad villus (bifid arrow), and crypts (C) with many goblet cells (arrowheads) ((**b**): H&E, ×100, Scale bar: 100 µm., C: ×400, Scale bar: 20 µm. (**a**,**d**–**g**): ×200, Scale bars: 50 µm).

**Figure 5:**
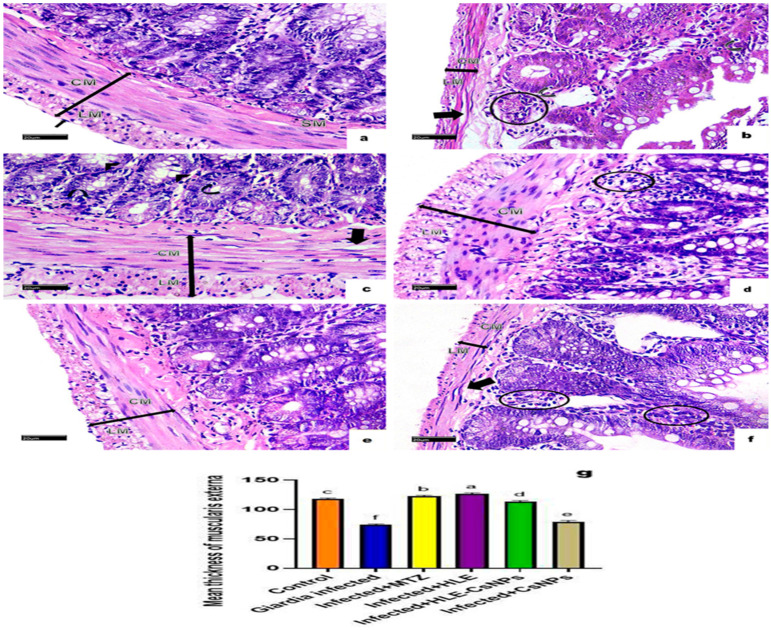
Light photomicrographs of sections of the small intestine of rats stained with H&E in all experimental groups. (**a**) The control group (non-infected non-treated) shows normal submucosa (SM) and muscularis externa (double-headed arrow) that consists of inner circular (CM), outer longitudinal smooth muscles (LM), and serosa (arrow). (**b**) The *Giardia*-infected group (infected non-treated) shows an apparently thin (double-headed arrow) muscularis externa (CM + LM) with disrupted circular smooth muscle cells (thick arrow). Some cells in the crypts and villi appeared with small dark nuclei (curved arrows). Mononuclear cellular infiltration is also noticed (circle). (**c**) The Infected + MTZ-treated group shows many goblet cells (arrowheads) and an apparently thick (double-headed arrow) muscularis externa (CM + LM) with vacuolated circular smooth muscle cells (thick arrow). Some cells lining the crypts appear with small dark nuclei (curved arrows). (**d**) Infected + HLE-treated group, there is an apparently thick (double-headed arrow) muscularis externa (CM + LM). Mild mononuclear cellular infiltration in the submucosa (circle) is also noticed. (**e**) The infected + HLE-CsNPs-treated group, there is an apparently thick (double-headed arrow) muscularis externa (CM + LM). (**f**) The infected + CsNPs-treated group, there is an apparently thin (double-headed arrow) muscularis externa (CM + LM) with disrupted circular smooth muscle cells (thick arrow). Moderate mononuclear cellular infiltration (circles) is observed. (**g**) Histomorphometry measures show the mean ± SD of the thickness of the muscularis externa in the experimental and treated groups. One-way ANOVA test was used for analysis, followed by Tukey’s post hoc comparison test, at *p* < 0.05. Group comparison is demonstrated in the bar chart: common letters indicate non-significant means, while different letters indicate statistically significant means. (**a**–**f**): (H&E ×400, Scale bar: 20 µm).

H&E-stained sections from the small intestines of the non-infected, non-treated group displayed a normal structure, consisting of the mucosa, submucosa, muscularis externa, and serosa. The mucosa featured finger-like villi lined with simple columnar epithelium, which included enterocytes and goblet cells. Enterocytes had oval basal nuclei and acidophilic cytoplasm, while intestinal glands, known as the crypts of Lieberkühn, were present between the villi. The submucosa contained loose connective tissue, and the muscularis externa was made up of an inner circular layer and an outer longitudinal layer (Figure 4a and Figure 5a). In the infected, non-treated group, the intestinal architecture was disrupted. The villi were shortened, broad, and some were fused, accompanied by ulcerations and atrophy. Inflammatory infiltrations and altered goblet cells were observed, as well as a thinner muscularis externa (Figure 4b,c and Figure 5b). In animals from the group infected and treated with MTZ, moderate improvements were observed, including increased goblet cells and disrupted muscularis externa (Figure 4d and Figure 5c). Animals in the groups infected and treated with HLE and HLE-CsNPs showed significant recovery, with restored villi and a thickened muscularis externa (Figure 4e,f and Figure 5d,e). However, animals in the group treated with CsNPs exhibited moderate inflammation and a thinner muscularis externa compared to the other groups (Figure 4g and Figure 5f). Morphometric analysis revealed a significant decrease in the thickness of the muscularis externa in the infected group compared to the non-infected, non-treated group. Conversely, the infected groups treated with MTZ, HLE, and HLE-CsNPs showed significant increases in muscularis externa thickness. The infected group treated with CsNPs, however, had a significantly thinner muscularis externa compared to the others (Figure 5g). Preliminary analysis indicated no significant differences in muscularis externa thickness or mean area percentages of PAS and CD117 reactions among the non-infected non-treated and the non-infected treated groups (I and VII–X). Therefore, their data were combined into a single control group for statistical comparisons.

#### 3.5.2. PAS Reaction-Stained Sections

**Figure 6:**
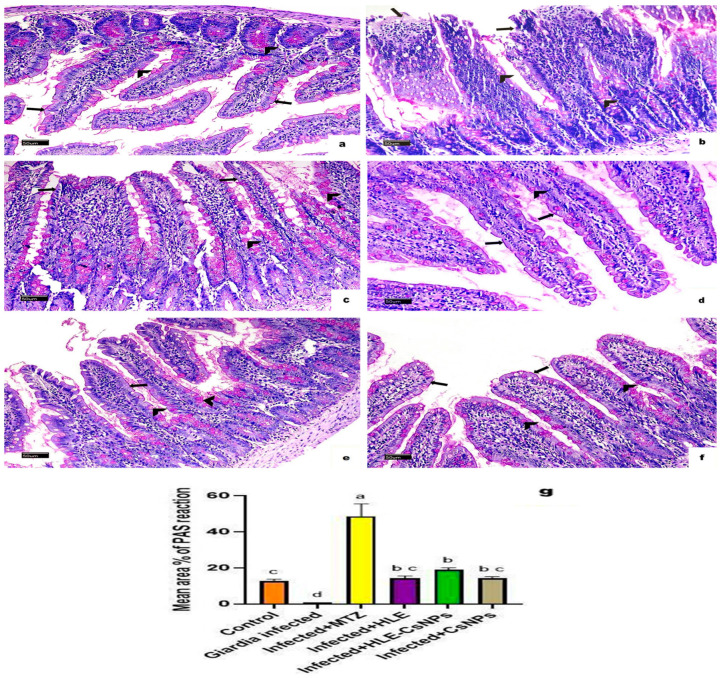
Light photomicrographs of a section of the small intestine of rats with PAS reaction in all experimental groups. (**a**) The control group (non-infected non-treated) shows intense positive PAS reaction in goblet cells (arrowheads) and brush border (arrows). (**b**) The *Giardia*-infected (infected non-treated) group shows a mild reaction in goblet cells (arrowheads) and a negative reaction in the intestinal brush border (arrows). (**c**) The infected + MTZ-treated group shows intense positive PAS reaction in goblet cells (arrowheads) and mild reaction at the brush border of some villi (arrows). (**d**–**f**) The infected, infected + HLE-CsNPs, and infected + CsNPs treated groups, respectively, show moderate positive PAS reaction in goblet cells (arrowheads) and brush border (arrows). (**g**) Histomorphometry measures show the mean ± SD of the area percentage of PAS reaction in the small intestine of the experimental and treated groups. One-way ANOVA test was used for analysis, followed by Tukey’s post hoc comparison test, at *p* < 0.05. Group comparison is demonstrated in the bar chart: common letters indicate means that are nonsignificant, while different letters indicate statistically significant means. ((**a**–**f**): PAS reaction ×200, Scale bar: 50 µm).

Sections of the small intestine displayed a strong PAS-positive reaction in goblet cells in the non-infected, non-treated group (Figure 6a). In the infected, non-treated group, goblet cells showed a mild reaction, and there was no reactivity observed in the intestinal brush border (Figure 6b). In contrast, the infected group that received MTZ treatment exhibited an intense reaction in goblet cells (Figure 6c). Moderate reactions were noted in the infected groups treated with HLE, HLE-CsNPs, and CsNPs (Figure 6d–f). Quantitatively, the infected non-treated group showed a significant decrease in the mean area percentage of PAS reaction compared to the non-infected, non-treated group (*p* < 0.05). Conversely, the infected group treated with MTZ exhibited a highly significant increase in the mean area percentage compared to the non-infected, non-treated group (*p* < 0.05). Additionally, the mean area percentage of PAS reaction was significantly higher in the groups infected and treated with HLE, HLE-CsNPs, and CsNPs compared to the infected non-treated group (*p* < 0.05), although it remained significantly lower than that observed in the MTZ-treated infected group (*p* < 0.05) (Figure 6g).

#### 3.5.3. Immunohistochemical Reaction for CD117/c-Kit Antibody

**Figure 7:**
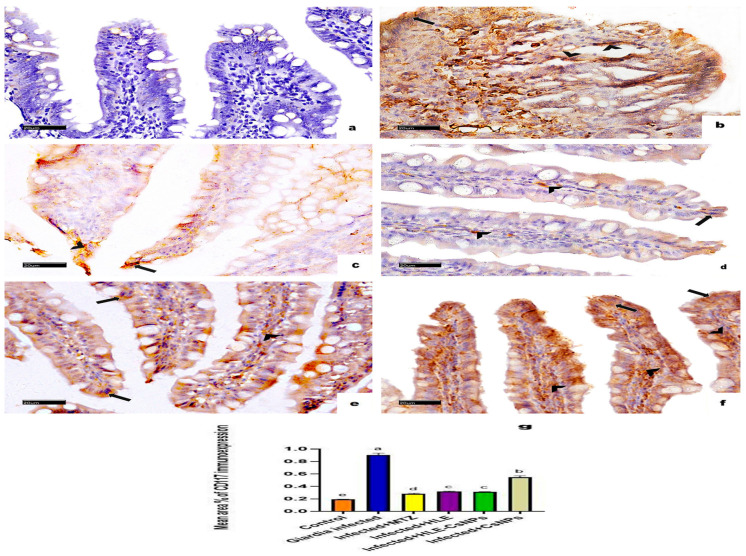
Immune-light photomicrographs of CD117/c-Kit immunohistochemically stained sections in the small intestine of rats in all experimental groups. The positive reaction appears as brown cytoplasmic discoloration (arrowheads in the cells of CT corium and arrows in the enterocytes). (**a**) The control group (non-infected non-treated) shows diffuse negative CD117 immunoreaction. (**b**) The *Giardia*-infected group (infected non-treated) exhibits marked CD117 cytoplasmic immunoreactivity in the enterocytes and cells of the CT corium. (**c**–**e**) Infected + MTZ, Infected + HLE, and Infected + HLE-CsNPs treated groups, respectively, show mild cytoplasmic immunoreaction in the enterocytes and the cells of CT corium. (**f**) The infected + CsNPs-treated group shows moderate CD117 cytoplasmic immunoreaction in the enterocytes and the cells of CT corium. (**g**) Histomorphometric measures show the mean ± SD of the area percentage of CD117 immunoreaction in the small intestine of the experimental and treated groups. One-way ANOVA test was used for analysis, followed by Tukey’s post hoc comparison test, at *p* < 0.05. Group comparison is demonstrated in the bar chart: common letters indicate means that are not statistically significant, while different letters indicate statistically significant means. ((**a**–**f**): CD117/c-Kit immunohistochemical reaction ×400, Scale bar: 20 µm).

CD117/c-Kit immunohistochemical staining showed no reactions in the non-infected, non-treated group (Figure 7a). In contrast, the infected, non-treated group displayed significant cytoplasmic immunoreactivity for CD117 in both the enterocytes and the corium cells (Figure 7b). The infected groups treated with MTZ, HLE, and HLE-CsNPs exhibited mild cytoplasmic immunoreactivity (Figure 7c–e). Additionally, the infected group treated with CsNPs showed moderate cytoplasmic immunoreactivity in the enterocytes and corium cells (Figure 7f). Quantitatively, the mean area percentage of CD117 immunoreactivity in the infected, non-treated group was significantly higher than in the non-infected, non-treated group. Moreover, all infected and treated groups demonstrated a significant increase in immunoreactivity compared to the non-infected, non-treated group (*p* < 0.05), but they showed a significant decrease compared to the infected, non-treated group (*p* < 0.05) (Figure 7g).

## 4. Discussion

*Giardia duodenalis* is one of the most common intestinal parasites and the leading protozoal cause of gastroenteritis worldwide. The standard treatment for *G. duodenalis* infection is metronidazole (MTZ), despite its known serious side effects and the risk of developing resistance in the parasite [55,56]. As a result, significant efforts have been made to identify new, alternative, effective, and safe anti-giardiasis drugs. The use of herbs and medicinal plants to eliminate *Giardia duodenalis* cysts and trophozoites has recently been considered a safer alternative to traditional methods [57]. Additionally, incorporating nanoparticles can help reduce adverse effects and improve the pharmacokinetic parameters of many herbal treatments [58]. Therefore, this study aimed to evaluate the therapeutic effects of *Hedera helix* leaf extract and *Hedera helix* leaf extract-loaded chitosan nanoparticles (HLE-CsNPs) compared with metronidazole (MTZ) for treating *Giardia duodenalis* isolated from the stool of individuals with gastrointestinal issues, using an experimental rat model.

This study identified *Giardia duodenalis* assemblage B as the only genotype among infected individuals, with a prevalence of 4.8% in patients with gastrointestinal complaints. This particular genotype is widely linked to human giardiasis worldwide [59]. Assemblage B is notable for its diverse range of host species, its potential for zoonotic transmission, and its frequent association with symptomatic infections, making it especially relevant for experimental modeling [11,60]. Detecting assemblage B in the studied population underscores the significance of the experimental giardiasis model used in this research for evaluating the effectiveness of *Hedera helix* extract-loaded chitosan nanoparticles as a treatment. By focusing on a clinically important and pathogenic assemblage, the experimental model more accurately reflects the dynamics of human infections and reinforces the biological rationale for exploring alternative therapeutic strategies [47].

In our study, inoculation of *Giardia duodenalis* in the non-treated group induced intestinal tissue toxic effects, characterized by severe villous atrophy, ulceration, and loss of the brush border, which coincided with earlier studies demonstrating giardiasis-induced tissue damage [61,62]. This was explained by the release of parasite proteins characterized by proteolytic activity, which cause enterocyte destruction and disruption of cell–cell connections, resulting in atrophic tissue alterations [63]. Additionally, massive infiltration of the lamina propria with inflammatory cells, mainly lymphocytes and eosinophils, was observed, associated with apoptotic cells with pyknotic nuclei. Similar observations were reported in an earlier study conducted by Ventura et al. [64]. These findings can be explained by increased rates of enterocyte apoptosis with disruption of intestinal barrier function and mechanisms involved in the pathophysiology of giardiasis-associated acute diarrhea. Moreover, caspase-dependent apoptosis signaling pathways can participate in the pathogenesis of giardiasis, as previously suggested [65]. Our results also showed a significant reduction in the thickness of the muscularis externa compared to other groups and disruption of muscle cells in the infected non-treated group. These findings are in line with previous reports [66,67]. Additionally, alterations in the structure of the crypts were previously reported by Doğan et al. [68]. Furthermore, Pavanelli et al. [66] showed that enteric neurons and smooth muscles are affected, leading to a reduction in the thickness of the muscularis externa of the intestine. Interestingly, Abd-Elhamid et al. [67] and El-Gendy et al. [47] have shown that infection with *G. lamblia* leads to goblet cell depletion. Similarly, by measuring the area percentage of PAS reaction, our results showed that the *Giardia*-infected group demonstrated a significant reduction in mucus secreted by goblet cells compared to the non-infected and the infected treated groups. Amat et al. [69] attributed the giardiasis-induced reduction in goblet cells to the breakdown of mucin by trophozoites’ proteases. Additionally, giardiasis causes depletion of mucin in the host intestine through a combination of mucus breakdown and hypersecretion by goblet cells.

The tested treatments in the current study resulted in a significant reduction in infection density, measured by the number of cysts per gram of stool, compared to the untreated groups. The maximal improvement was observed in the infected + MTZ-treated group, followed by the infected + HLE-CsNPs, then the infected + HLE-treated group. The least improvement was detected in the infected + CsNPs-treated group; however, it still showed statistically significant improvement compared to the untreated groups. The infected group treated with MTZ showed partial improvement and healing of intestinal tissues, detected by mild shortening and broadening of villi and decreased inflammatory cellular infiltrations. In addition to the preserved ultrastructural changes, the mean thickness of the muscularis externa was significantly increased compared to the infected, untreated group. These results agree with Mazroue et al. [70], who observed partial healing of intestinal villi after MTZ treatment in experimental *Giardia duodenalis* infection. Additionally, decreased cellular infiltration of the lamina propria was noticed in this group. This may be due to the antioxidant, anti-inflammatory, and immunomodulatory effects of MTZ [47]. Measuring the area percentage of PAS reaction was used to assess goblet cell secretion, revealing a significant increase in goblet cell mucus compared to the infected, non-treated, and non-infected non-treated groups. This finding aligns with Pelissier et al. [71], who reported that MTZ treatment results in thickening of the mucus layer in the proximal colon of rats. Sections from the infected + MTZ-treated group showed a highly significant decrease in area percentage of CD117 immunoreactivity due to the anti-*Giardia* effect of MTZ, as previously demonstrated by Harba et al. [72].

In the current study, histopathological examination of the infected + HLE, infected + HLE-CsNPs, and infected + CsNPs-treated groups showed remarkable improvement compared to infected nontreated groups in the form of restoration of villus and goblet cells configuration with mild cellular infiltrations. Moreover, restoration of goblet cell number and increased mucus secretion were detected in the infected + HLE-treated group and confirmed by measuring PAS area percentage. This agrees with Shokry et al. [73], who reported that both ethanolic HLE and saponin-rich fractions significantly suppressed inflammatory cells and reduced goblet cells metaplasia. Furthermore, Shokry et al. [51] added that HLE is a rich source of phytochemicals with demonstrated therapeutic activities commonly used in folklore as inhibitors of inflammation, bronchodilators, analgesics, anticancer, antimicrobial, and anticoagulant. They can also treat protozoal, bacterial, and fungal infections. This could be attributable to the direct cytotoxic effect of HLE on trophozoites, as it also significantly decreases CD117 compared to infected untreated groups. Sinelnikov et al. [13] proved that CD117/C-kit immunostaining is useful for the diagnosis of *G. duodenalis* in duodenal biopsies. The beneficial effects of HLE are consistent with those of Shokry et al. [73], who recommended using standardized HLE for its anti-inflammatory, antioxidant, and antimicrobial activities. Moreover, Schulte-Michels et al. [74] recognized that the favorable effects of HLE are due to its flavonoid content, which has anti-inflammatory potential.

Histopathological results from the infected + HLE-CsNPs and infected + CsNPs-treated groups showed improvement in villous and crypt structures with decreased cellular infiltrations and improved goblet cell secretion associated with reduced inflammatory cellular infiltration. These findings align with an earlier study by El-Gendy et al. [47]. This improvement was most prominent in the infected + HLE-CsNPs group, where the thickness of ME was significantly increased compared to the untreated group and not significantly different from the infected + MTZ-treated group. The infected + CsNPs-treated group showed a significant decrease in thickness compared to other groups. Additionally, goblet cell regeneration was significantly increased in infected + HLE-CsNPs and infected + CsNPs-treated groups compared to infected untreated groups. Moreover, Said et al. [75] recognized the efficacy of CsNPs against *Giardia* trophozoites, explaining it by their mucoadhesive properties that prolong the time of action and reduce elimination in the gastrointestinal tract. Additionally, Kim [76] proved that CsNPs have anti-inflammatory, anti-microbial, and antioxidant effects. As a result, the area percentage of CD117 significantly decreased in the infected + HLE-CsNPs group more than in the infected + CsNPs-treated groups compared to the untreated group. This demonstrates the higher efficiency of HLE-CsNPs compared to CsNPs. On the other hand, MTZ treatment was associated with significant liver and kidney organ stress, identified by increased serum GOT, GPT, urea, and creatinine in both infected and non-infected groups treated with MTZ. Conversely, other treatments, HLE, HLE-CsNPs, and free CsNPs, did not cause an increase in SGOT, SGPT, serum urea, or creatinine levels. The effectiveness of numerous herbal extracts against *Giardia* infection was demonstrated in earlier studies. For example, Pintong et al. [77] observed that white-purple leaf and flower purple extracts can treat *G. duodenalis* trophozoites, attributing this to their ability to induce degeneration of flagella and ventral discs of *G. duodenalis* trophozoites. Azadbakht et al. [78] recognized that the hydroalcoholic extract of *Artemisia annua* can inhibit *G. duodenalis* cysts in vitro. Harba et al. [72] reported inhibitory effects of *Cymbagogon citratus* aqueous extracts both in vitro and in vivo against *G. lamblia*. In addition to those of El-Shennawy et al. [79], a significant reduction in *Giardia* was noted due to pomegranate peel extract treatment.

The negative impact of MTZ on the liver and kidneys identified in this study is consistent with observations from earlier studies [80,81,82], where the hepatotoxic risk of MTZ was recognized and explained by its metabolites, which bind to RNA instead of DNA, inhibiting RNA protein synthesis. Originally, MTZ was thought to interact with DNA, causing disruption of DNA structure and inhibition of protein synthesis, leading to cell death. A similar mechanism may explain the MTZ-associated renal stress identified in the current study and reported earlier [83]. On the other hand, normal liver and renal function tests in the infected + HLE, infected + HLE-CsNPs, and infected + CsNPs-treated groups suggest their protective effect against liver and kidney damage. This may be attributed to the anti-inflammatory and antioxidant properties of HLE [84,85]. Notably, a recent study conducted by Shokry et al. [73] observed that bioactive phenolic content in HLE could reduce inflammatory cytokines and oxidative stress biomarkers, minimizing liver injury in an in vivo acute lung inflammation model. Additionally, chitosan has biological pharmacological actions, including antioxidants, bacteriostatic, immunomodulatory, and anticancer properties. Furthermore, the antimicrobial properties of chitosan are due to its interaction with anionic molecules on the cell surface. Moreover, the suggested renoprotective action of HLE may be due to the effect of alkaloids and triterpene saponins in *Hedra helix* leaves, which are effective against intestinal parasites and fungal infections [51]. Additionally, HLE components, especially rutin, quercetin, saponins, and flavonoids, have demonstrated nephroprotective effects. Similarly, hederagenin, a monomer compound in *Hedra helix*, significantly decreased serum creatinine and urea nitrogen levels in a cisplatin-induced acute kidney injury mouse model. These findings explain why the improvement in the infected + HLE-CsNPs-treated group was better than in the infected + CsNPs-treated group. While chitosan alone has anti-inflammatory and antimicrobial effects, its efficacy is enhanced when used as a nanoparticle carrier for other drugs, improving bioavailability and efficacy against target organisms [30,51,73]. Similarly, chitosan has been shown to exhibit antioxidant, bacteriostatic, immunomodulatory, and anticancer properties, which may contribute to the tissue-protective effects observed in this study [76,86]. Additionally, its pH-responsive solubility and mucoadhesive properties enable opening of tight junctions, facilitating enhanced drug delivery. Moreover, chitosan’s antimicrobial activity, attributed to its interaction with anionic molecules on the cell surface, has been recently reported by Yan et al. [87]. Overall, the findings of this study highlight the promising antigiardial activity and organ protection of HLE and HLE-CsNPs against experimentally induced giardiasis.

## 5. Conclusions

This study found that *Hedera helix* leaf extract (HLE) and HLE-loaded chitosan nanoparticles (HLE-CsNPs) effectively protect the liver and kidneys while reducing *Giardia duodenalis* infection in rats. Given the side effects and resistance issues with metronidazole, HLE-CsNPs present a potential alternative treatment option for giardiasis, particularly for those with gastrointestinal symptoms and zoonotic concerns.

## Figures and Tables

**Table 1:** Impact of treatment on *G. duodenalis* cyst count per gram of feces and the percentage reduction in infection.

Studied Groups	Cysts in One Gram of Feces	Reduction Rate %
Infected non-treated	92,853 ^a^ ± 79.2 (3431.9)	-
Infected + MTZ-treated	762 ^b^ ± 15.1 (134.6)	99.17%
Infected + HLE-treated	19,736 ^c^ ± 33.4 (13,716.9)	78.7%
Infected + HLE-CsNPs-treated	4437 ^b^ ± 24.1 (1435.3)	88.8%
Infected + CsNPs-treated	27,991.8 ^c^ ± 41.8 (12,156.6)	69.8%
F	19.886 *	
P	<0.001 *	

Data are analyzed using a one-way ANOVA test followed by post hoc Tukey’s test and presented as mean ± SD (n = 12 rats/group). P: *p*-value for comparing between the studied groups: *: Statistically significant at *p* ≤ 0.05. Means with common letters are not significant, while means with different letters are significant.

**Table 2:** Effect of treatments on renal and liver function tests.

Studied Groups	SGPT (U/L)	SGOT (U/L)	Creatinine (mg/dL)	Urea (mg/dL)
Non-infected non-treated	114 ^b^ ± 13	126.3 ^b^ ± 10.4	0.21 ^b^ ± 0.01	28.5 ^b^ ± 5.5
Infected non-treated	118.3 ^b^ ± 13.5	123.7 ^b^ ± 15	0.23 ^b^ ± 0.03	31.2 ^a^ ± 4.6
Infected + MTZ-treated	144 ^a^ ± 9.3	150 ^a^ ± 18.7	0.35 ^a^ ± 0.04	32.7 ^a^ ± 8.5
Infected + HLE-treated	117.5 ^b^ ± 10.4	133.2 ^a,b^ ± 5.1	0.28 ^a,b^ ± 0.13	25.5 ^c^ ± 3.7
infected + HLE-CsNPs-treated	118.5 ^b^ ± 6.1	128.3 ^b^ ± 11.5	0.23 ^b^ ± 0.02	23.2 ^c^ ± 4.6
Infected + CsNPs-treated	124.5 ^b^ ± 5	122.2 ^b^ ± 7.9	0.25 ^b^ ± 0.04	32.2 ^a^ ± 1.7
Non-infected+ HLE-treated	114.2 ^b^ ± 8.6	126.7 ^b^ ± 12.7	0.22 ^b^ ± 0.01	26.7 ^b,c^ ± 5.2
Non-infected + HLE-CsNPs treated	116.5 ^b^ ± 11.1	120.5 ^b^ ± 9.8	0.23 ^b^ ± 0.03	25.3 ^c^ ± 1.2
Non-infected + CsNPs-treated	129 ^a,b^ ± 6.7	130.8 ^a,b^ ± 5.5	0.25 ^a,b^ ± 0.04	31.8 ^a^ ± 6.3
Non-infected + MTZ-treated	132.2 ^a,b^ ± 8.7	140.7 ^a,b^ ± 3.3	0.28 ^a,b^ ± 0.06	34.7 ^a^ ± 5.4
F	6.025 *	4.118 *	3.605 *	2.354 *
P	<0.001 *	0.001 *	0.002 *	0.027 *

Data are analyzed using a one-way ANOVA test followed by post hoc Tukey’s test and presented as mean ± SD (n = 12 rats/group). P: *p*-value for comparing between the studied groups: *: Statistically significant at *p* ≤ 0.05. Means with common letters are not significant, while means with different letters are significant.

## Data Availability

The original contributions presented in this study are included in the article. Further inquiries can be directed to the corresponding author.

## Supplementary Materials

The following supporting information can be downloaded at: https://www.mdpi.com/article/10.3390/vetsci13020207/s1, Figure S1: Agarose gel electrophoresis illustrating nested-PCR-RFLP results of Giardia duodenalis characterization; Table S1: Nested PCR amplification reaction conditions; Table S2: Prevalence of Giardia duodenalis infections and the assemblages identified from stool samples of surveyed individuals (n = 147).
