# Supplementary material for: Antiparasitic Activity of Hedera helix Extract-Loaded Chitosan Nanoparticles in Experimentally Induced Giardiasis
Source: Vet Sci. 2026 Feb 22;13(2):207. doi: 10.3390/vetsci13020207 (PMC12944906; doi:10.3390/vetsci13020207)
Supplement: Supplementary file 1 [file vetsci-13-00207-s001.zip › Supplementary figure.pdf]

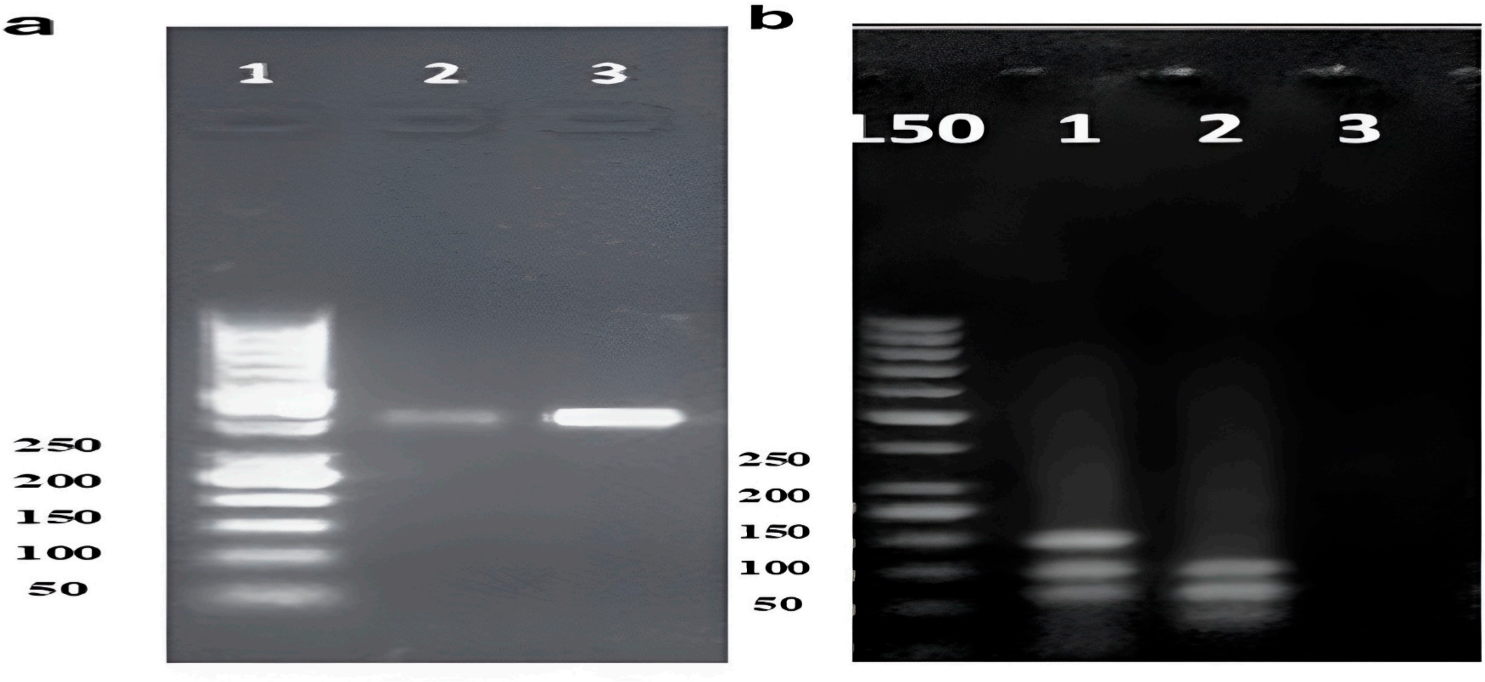

Figure S1. Agarose gel electrophoresis illustrating nested-PCR-RFLP results of *Giardia duodenalis* characterization. The target gene was *B-giardin*. a) Lane 1 (L1): a DNA marker, L2: *Giardia* assemblage B digestion product. b) L50: a 50-bp DNA MM marker; L1: positive control samples of assemblage A showing a faint 50-bp band, 110, 150, and 210 bp bands; L2: Positive control samples of assemblage B showing a faint 24/26-bp band, 84, 117, and 150 bp; L3: negative control samples.
