# Supplementary material for: Antiparasitic Activity of Hedera helix Extract-Loaded Chitosan Nanoparticles in Experimentally Induced Giardiasis
Source: Vet Sci. 2026 Feb 22;13(2):207. doi: 10.3390/vetsci13020207 (PMC12944906; doi:10.3390/vetsci13020207)
Supplement: Supplementary file 1 [file vetsci-13-00207-s001.zip › Supplementary tables.pdf]

Table S1. Nested PCR amplification reaction conditions

	1st amplification reaction	2nd amplification reaction
**Generated fragment length**	753bp	511bp
**Primer pair sequence**	G7: 5'-AAG CCC GAC GAC CTC ACC CGC AGT GC -3'<br>G759: 5'-CAT AAC GAC GCC ATC GCG GCT CTC AGG AA-3'	2005F; 5'- GAA C GA ACG AGA TCG AGG TCC G -3'<br>2005R; 5'-CTC GAC GAG CTT CGT GTT-3'
**Denaturation**	35 cycles at 95ºC for 30 sec	35 cycles at 95ºC for 30 sec
**Annealing**	at 65ºC for 30 sec	at 53ºC for 30 sec
**Extension**	at 72 ºC for 10 min	at 72 ºC for 10 min

Table S2: Prevalence of *Giardia duodenalis* infections and the assemblages identified from stool samples of surveyed individuals (n = 147)

Factors		*G. duodenalis* No. Positive/No. examined (%)	Assemblage(s) (n)
Age (Years)	10-30	4/80 (5.0)	B (4)
	31-60	3/67 (4.5)	B (3)
Sex	Male	3/69 (4.3)	B (3)
	Female	4/78 (5.1)	B (4)
Symptoms	Diarrhea	2/42 (4.8)	B (2)
	Non- diarrhea	5/105 (4.8)	B (5)
Total		7/147 (4.8)	B (7)
